# The Potential of Lonidamine in Combination with Chemotherapy and Physical Therapy in Cancer Treatment

**DOI:** 10.3390/cancers12113332

**Published:** 2020-11-11

**Authors:** Yaxin Huang, Guohui Sun, Xiaodong Sun, Feifan Li, Lijiao Zhao, Rugang Zhong, Yongzhen Peng

**Affiliations:** 1 Beijing Key Laboratory of Environment and Viral Oncology, College of Life Science and Chemistry, Faculty of Environment and Life, Beijing University of Technology, Beijing 100124, China; huangyaxin@emails.bjut.edu.cn (Y.H.); sunxd@emails.bjut.edu.cn (X.S.); lifeifan@emails.bjut.edu.cn (F.L.); zhaolijiao@bjut.edu.cn (L.Z.); lifesci@bjut.edu.cn (R.Z.); 2 National Engineering Laboratory for Advanced Municipal Wastewater Treatment & Reuse Technology, Engineering Research Center of Beijing, Beijing University of Technology, Beijing 100124, China; pyz@bjut.edu.cn

**Keywords:** Lonidamine, energy metabolism, combination, resistance, chemotherapy, physical therapy

## Abstract

**Simple Summary:**

The unique characteristics of tumor energy metabolism (highly dependent on aerobic glycolysis, namely, the Warburg effect) make it an interesting and attractive target for drug discovery. Radio- and chemoresistance are closely associated with the Warburg effect. Lonidamine (LND), as a glycolytic inhibitor, although having low anticancer activity when used alone, exhibits selectivity to various tumors, and its adverse effects do not overlap when combined with other chemotherapeutic drugs. Therefore, LND may be very promising as a sensitizer of tumors to chemotherapeutic agents and physical therapies. This review summarizes the advance of LND in combination with chemotherapy and physical therapy over the past several decades, as well as the promising LND derivative adjudin (ADD). The underlying sensitizing mechanisms were also analyzed and discussed, which may contribute to an improved therapeutic effect in future clinical cancer treatment.

**Abstract:**

Lonidamine (LND) has the ability to resist spermatogenesis and was first used as an anti-spermatogenic agent. Later, it was found that LND has a degree of anticancer activity. Currently, LND is known to target energy metabolism, mainly involving the inhibition of monocarboxylate transporter (MCT), mitochondrial pyruvate carrier (MPC), respiratory chain complex I/II, mitochondrial permeability transition (PT) pore, and hexokinase II (HK-II). However, phase II clinical studies showed that LND alone had a weak therapeutic effect, and the effect was short and reversible. Interestingly, LND does not have the common side effects of traditional chemotherapeutic drugs, such as alopecia and myelosuppression. In addition, LND has selective activity toward various tumors, and its toxic and side effects do not overlap when combined with other chemotherapeutic drugs. Therefore, LND is commonly used as a chemosensitizer to enhance the antitumor effects of chemotherapeutic drugs based on its disruption of energy metabolism relating to chemo- or radioresistance. In this review, we summarized the combination treatments of LND with several typical chemotherapeutic drugs and several common physical therapies, such as radiotherapy (RT), hyperthermia (HT), and photodynamic therapy (PDT), and discussed the underlying mechanisms of action. Meanwhile, the development of novel formulations of LND in recent years and the research progress of LND derivative adjudin (ADD) as an anticancer drug were also discussed.

## 1. Introduction

**Figure 1:**
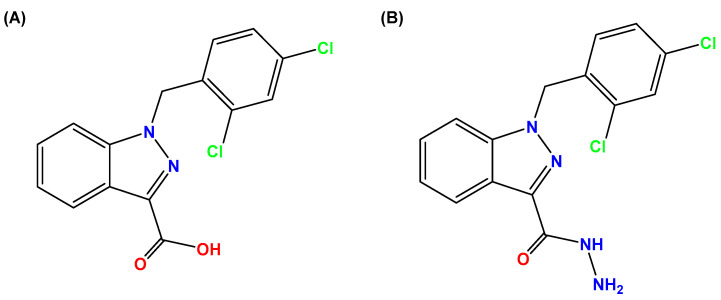
Molecular structures of (**A**) LND and (**B**) ADD. LND and ADD are indazole derivatives. Their chemical names are 1-(2,4-dichlorobenzyl)-1*H*-indazole-3-carboxylic acid and 1-(2,4-dichlorobenzyl)-1*H*-indazole-3-carbohydrazide, respectively.

**Figure 2:**
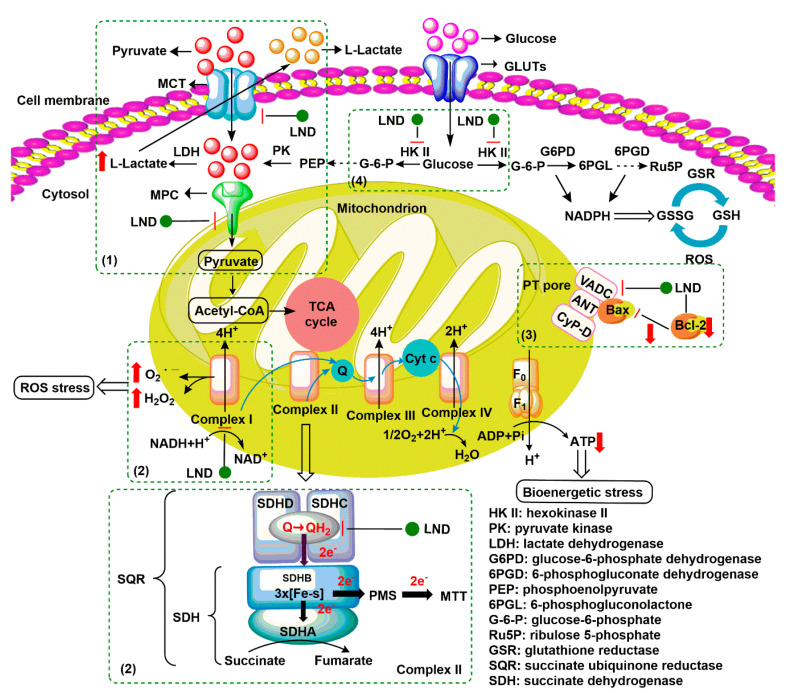
Underlying mechanisms of LND-mediated antitumor activity. (1) LND inhibits the lactate export and the uptake of pyruvate into mitochondria by the inhibition of proton-linked monocarboxylate transporter (MCT) and mitochondrial pyruvate carrier (MPC), respectively. (2) LND inhibits complexes I and II by interfering with ubiquinone reduction, leading to ATP depletion and ROS production. (3) LND affects the mitochondrial permeability transition (PT) pore which is under the control of the members of the Bcl-2 family. (4) LND inhibits glycolysis through the inhibition of hexokinase II (HK-II), thereby reducing the levels of NADPH and glutathione (GSH) in part by the inhibition of the pentose phosphate pathway (PPP) flux.

Lonidamine (LND) is an indazole derivative (Figure 1A) that was first introduced in 1979 as an anti-spermatogenic agent [1]. Later, LND was found to have antitumor activity by interfering with the energy metabolism, especially its action on tumor mitochondria. First, LND inhibits lactate export and the uptake of pyruvate into mitochondria by the inhibition of proton-linked monocarboxylate transporter (MCT) and mitochondrial pyruvate carrier (MPC), respectively. However, the IC_50_ of LND against MPC is an order of magnitude lower than the IC_50_ against MCT [2]. Therefore, LND-mediated inhibition of MPC is likely to play a key role. Second, LND inhibits complexes I and II of the mitochondrial electron transport chain [2]. By measuring the succinate dehydrogenase (SDH) activity in complex II and the succinate-ubiquinone reductase (SQR) activity (determined via the formation of ubiquinone by complex II), the inhibition of SQR activity was found to be much greater than that of SDH activity at all LND concentrations tested [3]. Therefore, LND is thought to inhibit complex II activity by interfering with the ubiquinone binding sites of succinate dehydrogenase C (SDHC) and succinate dehydrogenase D (SDHD) (Figure 2) [3]. The inhibition of mitochondrial complex II can induce reactive oxidative species (ROS) production to promote cell apoptosis. Third, LND disrupts the mitochondrial transmembrane potential by directly affecting the mitochondrial permeability transition (PT) pore which is under the control of the members of the Bcl-2 family [4]. After LND treatment, AKT phosphorylation also decreases, which promotes the transfer of p53 from cytoplasm to mitochondria, leading to cell apoptosis [5,6]. Furthermore, some studies also indicated that LND inhibited hexokinase II (HK-II), followed by the inhibition of glycolytic pathway, as well as pentose phosphate pathway (PPP), leading to the reduction of NADPH and GSH levels. Thus, it was usually used as a glycolytic inhibitor for antitumor research (Figure 2) [7,8]. By using ^31^P NMR technology, LND was shown to produce intracellular acidification and de-energization in vitro and in vivo, and it exhibited selectivity against tumors in vivo without obvious toxic effects on skeletal muscle and brain [9]. Indeed, Nath et al. further showed that LND selectively reduced tumor intracellular pH and ATP levels, and sensitized DB-1 melanoma xenografts to melphalan [10]. LND has also been demonstrated to induce a cytotoxic autophagic response in glioblastoma cells [6]. LND triggers late autophagy, which eventually leads to the transition from autophagy to apoptosis that occurred before phosphatidylinositol disappeared and p-AKT decreased [6]. Currently, LND has been explored for the treatment of non-small cell lung cancer (NSCLC) [11], breast cancer [12], colon cancer [13], astrocytoma [14], squamous cell carcinoma, human glioma, and so on [15]. The research on LND’s derivative, adjudin (ADD), is also worthy of attention (Figure 1B). Similarly, ADD was also originally used as an anti-spermatogenic agent [16]. It was not until 2013 that Xie et al. determined, for the first time, that ADD also had anticancer properties, and consequently, revealed its potential clinical utility as a chemotherapeutic agent [17]. In this study, ADD was proved to be effective for at least fifteen cancer cell lines [17]. When in combination with other chemotherapeutics, ADD also showed a potent synergistic anticancer effect [18,19]. ADD also causes mitochondrial dysfunction to interfere with energy metabolism and induce mitochondrial apoptosis pathway, thus triggering cell apoptosis and autophagy [17]. As ADD, a simple LND derivative, also shows potential anticancer effects, we will briefly summarize the drug combinations related to ADD in recent years as well.

As a single chemotherapeutic agent, LND was found to be ineffective or only mildly effective in preventing the growth of cancer cells in vivo and in vitro, because its anticancer effects are transient and reversible [20]. In the phase II study of oral LND, the most common side effects were myalgia, weakness, and lethargy, testicular pain was also observed [21]. Myalgia displayed dose-limiting toxicity, occurring at a dose of 300–400 mg/m^2^ [22,23]. Severe vomiting and signs of acute hepatic and pancreatic toxicity were observed when the dose exceeded 400 mg/m^2^ during intravenous administration [24]. However, if receiving oral LND, it will face the problem of poor bioavailability.

Therefore, in order to reduce the toxic and side effects of LND by intravenous administration, it can be used in combination with other chemotherapeutic agents or physical therapies, or be loaded into a system with tumor targeting properties to reduce the damage to normal cells [24,25,26]. Interestingly, LND has no common side effects of traditional anticancer drugs, such as bone marrow suppression, alopecia, gastrointestinal mucosal necrosis, and does not cause somatic and germ cell mutations [21]. Moreover, because the association of energy metabolism of tumor cells with chemo- or radio-resistance [27], and LND-mediated inhibition of glycolysis and mitochondrial respiration [2], from the point of combination treatment, LND can potentiate the anticancer effects of chemotherapeutic drugs or enhance the therapeutic efficacy when in combination with physical therapies. In view of the tumor selectivity of LND, in this case, the dose of chemotherapeutic drugs may be decreased while avoiding or maximally decreasing the toxic and side effects. The most critical characteristic of LND is its selective activity against a wide range of tumors, with little effect on normal tissues at doses below 400 mg/m^2^ (oral or intravenous) [10]. Tumor selectivity and low toxicity to normal tissues are key features that make LND an attractive sensitizer to enhance the anticancer activity of chemotherapeutic drugs and physical therapies.

In addition to being a chemosensitizer, in the past five years, LND has often been coupled with targeted agents or other chemotherapeutics and encapsulated in a nanometer system to better improve its tumor targeting and the effectiveness of combined drugs [25,28,29]. For instance, in 2019, Cheng et al. developed the mitochondria-targeted LND (Mito-LND) by conjugating LND to triphenylphosphonium cation (TPP^+^) via a linker aliphatic chain with reference to other mitochondrial targeting agents such as Mito-Q and Mito-Metformin using Co-Q and metformin as bioactive molecules, respectively. Compared to free LND, Mito-LND not only had significantly higher cytotoxicity to lung cancer cells, but also showed inhibition of mice tumor xenografts and lung cancer brain metastasis in vivo by the inhibition of mitochondrial bioenergetics, induction of ROS and mitochondrial oxidative stress, downregulation of the AKT/mTOR/p70S6K signaling pathway, and induction of cytotoxic autophagy [28]. Especially, Mito-LND caused cell death mainly by autophagy rather than caspase-dependent apoptosis, as indicated by the conversion of LC3-I to autophagic LC3-II, markedly increased autophagic vacuoles, and flow cytometric analysis. In tumor tissues from mice, Mito-LND-induced autophagy was also observed. Of note, Mito-LND showed no observable toxicity in mice even at doses up to 50 times (375 μmol/kg) the effective inhibitory dose (7.5 μmol/kg) when administered for eight weeks. This study suggests that preparing proper formulations of LND may also be promising for the clinical treatment of cancers. In this review, we mainly focus on the combination of LND with chemotherapies and physical therapies.

## 2. Combination of LND with Chemotherapeutic Drugs

The complex biological characteristics of cancer make it is extremely difficult to get a monofunctional drug with satisfactory therapeutic efficacy for most cancers [30]. It may be promising that combination treatment, in which two or more drugs with different mechanisms are used together, reduces the drug resistance and kill cancer cells effectively. The following section is an overview of the combined effects of LND with several anticancer drugs. For different types of chemotherapy drugs, the synergistic mechanisms of LND to improve cell killing are also analyzed and discussed (Table 1).

### 2.1. LND in Combination with Cisplatin

Cisplatin (CDDP) is a classical Pt(II) drug, which exerts its anticancer activity via inducing the formation of DNA cross-linking [32]. Long-term use of CDDP alone will result in adverse reactions and drug tolerance. In MCF-7 breast carcinoma cells, the CDDP cytotoxicity was increased by approximately 100 folds after exposure to LND for 24 h pretreatment and after 12 h post-treatment [33]. Later, Madrid et al. demonstrated that the combination therapy of LND and CDDP was more effective in inhibiting tumor growth of MX-1 breast cancer and A2780 ovarian cancer than CDDP alone [34]. On the one hand, breast cancer MX-1 was highly sensitive to the combination of LND and CDDP, which was mainly because both CDDP and LND could induce the phosphorylation of Bcl-2 protein, and the antiapoptotic activity of tumor cells was decreased under the synergistic effect [34]. The synergy may be related to specific interference by LND in energy-dependent DNA repair process following the formation of CDDP-induced DNA damages. LND is an important sensitizing component of a combined regimen if the repair of a cytotoxic drug-induced damage is an energy-dependent process. LND alone only has a limited efficacy; however, when it was used as a chemosensitizer in combination with CDDP, it enhanced the efficacy of CDDP to tumors with a better response. In an early study, when LND was administrated at a later time after CDDP treatment, the cell killing decreased gradually, which was further consistent with the hypothesis that LND interfered with DNA repair mechanism [35]. In a clinical trial, Yu et al. used LND tablets combined with CDDP to treat patients with advanced NSCLC; the results showed that the combination of LND and CDDP had a synergistic effect on NSCLC patients, and could improve the life quality of patients compared to CDDP alone [36]. In addition to classical Pt(II) drugs (e.g., CDDP), other platinum complexes also receive increasing attention. Due to the unique inert physicochemical properties of Pt(IV) complexes, they exhibit fewer adverse reactions with biomolecules than Pt(II) species [70]. These low toxic or non-toxic Pt(IV) complexes can be activated by biological reductants, such as ascorbic acid or GSH, releasing toxic Pt(II) species [71]. Chen et al. developed several Pt(IV) prodrugs conjugated with sensitizer LND, of which, the CDDP-derived complex Pt(NH_3_)_2_(LND)Cl_3_ exhibited significantly increased anticancer activities against prostate adenocarcinoma LNCaP cells with nearly twice higher potency than CDDP and much more stronger than the physical mixture of CDDP and LND [72]. The Pt(IV) prodrugs conjugated with ADD (LND derivative) were also designed and determined their activities against CDDP-resistant triple-negative breast cancer [18]. These Pt(IV) prodrugs have self-assembly property to form nanoparticles. Yang et al. found that from the six synthesized Pt(IV) prodrugs, the most suitable C_4_-Pt-ADD or C_6_-Pt-ADD (ADD monosubstituted with butyl or hexyl contralateral substituted prodrug, respectively) assembled nanoparticles significantly enhanced the sensitivity of triple-negative breast cancer MDA-MB-231 cells to CDDP with approximately 266-fold lower IC_50_ value, significantly suppressed the tumor growth in vivo, and showed no significant organ (liver and kidney) damage compared to CDDP alone [18].

### 2.2. LND in Combination with Temozolomide

Temozolomide (TMZ) is a second-generation DNA alkylating agent, especially used for the treatment of gliomas because its ability to penetrate blood–brain barrier (BBB). It can spontaneously and rapidly degrade in vivo to produce the active mitozolomide (MTIC), which is converted to methyl diazonium ion, followed by causing DNA alkylation, leading to cell apoptosis [38]. TMZ is a first-line oral chemotherapeutic drug endorsed by the US Federal Drug Administration (FDA), but its efficacy is only about 45% due to the presence of tumor resistance [73]. Thus, it is rational to use a sensitizer to decrease the resistance, thereby increasing the anticancer activity of TMZ. TMZ is stable in acids, but it decomposes to MTIC by a base-catalyzed ring-opening reaction, then being converted to methyl diazonium ion, a potent alkylating species that preferentially interacts with the O6 or N7 site of guanine, resulting in DNA alkylation. As LND causes intracellular acidification through the inhibition of energy metabolism, it was expected that LND would increase the TMZ stability within tumors [38]. In a recent study, Nath et al. treated DB-1 human melanoma xenograft with LND in combination with TMZ, in which TMZ was injected 40 min after LND administration. Results showed that LND actually potentiated the short-term or long-term activity of TMZ to DB-1 human melanoma xenografts in mice [39]. At present, it is believed that the synergistic effect may be explained by the fact that LND-induced tumor de-energization inhibits the efflux of TMZ induced by energy-dependent multidrug resistance (MDR).

### 2.3. LND in Combination with Nitrogen Mustards

Nitrogen mustard (NM) is the first anticancer chemotherapeutic drug used in cancer treatment, which is derived from the military use of poison gas “mustard gas” in the First World War [74]. After entering the cells, NM can form electron-deficient ethyleneimine ions, and then covalently bind the nucleophile groups (electron-rich) of biomacromolecules for alkylation, resulting in DNA intrastrand and interstrand cross-linking [40]. In this case, the cells will die due to the inhibition of cell mitotic division. Due to the high level of aerobic glycolysis, tumors are typically characterized by a slightly acidic extracellular pH (pHe) and a neutral to alkaline intracellular pH (pHi) [75]. The typical pHe and pHi features affect tumor growth, metastasis, and therapeutic response. For instance, sodium bicarbonate can modify the tumor microenvironment by increasing pHe, thereby reducing tumor invasiveness [41]. Instead, the intracellular activity of nitrogen mustards (NMs) can be enhanced by lowering pHi, which stabilizes the aziridinium active intermediate and inhibits the activity of glutathione-*S*-transferase (GST) as a scavenger of aziridinium [42]. Therefore, intracellular acidifying agents may improve the therapeutic index of NMs by decreasing the pHi within tumor cells. LND inhibited the activity of MCT and MPC, which resulted in a significant increase in the total lactate level within tumor, most of which were trapped in the cytoplasm (pHi), although a slight decrease in pHe indicating the possible leak of lactate [43]. This was supported by Mardor et al.; they used diffusion-weighted imaging to demonstrate an increase in intracellular lactate in several human breast cancer and murine melanoma cell lines following LND treatment [44]. When melphalan was combined with LND pretreatment, the sensitivity of human DB-1 melanoma xenografts to melphalan was significantly increased with a growth delay of 19.9 days compared to melphalan alone of 4.0 days and LND alone of 1.1 days [42]. It should be noted that LND selectively reduced tumor pHi and bioenergetics, while no effects on the pHi or intracellular energy status (ATP/Pi) observed in the brain or skeletal muscle, and only a small transient decrease of pHi and energy in liver [43]. Because melphalan is a highly cytotoxic agent, to examine whether LND potentiates the anticancer activity of other NMs drugs, Nath et al. extended the NMs alkylating agents to chlorambucil, cyclophosphamide, and bendamustine [42]. They found that compared to the NMs alkylating agent alone, the combination of each NMs alkylating agent with LND produced significant or near significant tumor growth delays. Although the three NMs alkylating agents were less effective than melphalan when combined with LND for treating human DB-1 melanoma xenografts in mice, the systemic toxicity was relatively less serious [44]. For NMs alkylating agents, the mechanism of LND-induced potentiation may include (1) intracellular acid leads to increased concentration of the active aziridinium ion intermediate that yields DNA damage; (2) de-energization prevents the energy-dependent MDR pump from pumping out the drugs; (3) under acidic condition, GST activity is inhibited, leading to decreased level of GSH which can quench the active aziridinium species; and (4) reduced DNA repair due to the acid inhibition of repair protein [45,46]. In fact, this mechanism resembles the “HLAGR” mechanism recently proposed by us in the chemosensitization of carmustine (BCNU) mediated by another glycolytic inhibitor 3-bromopyruvate [76,77].

### 2.4. LND in Combination with Carmustine

Carmustine (BCNU) is a nitrosourea alkylating agent, which exerts its anticancer activity mainly through the O^6^-alkylation of DNA guanine, followed by the formation of DNA interstrand cross-linking [49]. However, O^6^-methylguanine-DNA methyltransferase (MGMT/AGT) can repair such DNA alkylation damage, thereby leading to tumor resistance [78]. In the early 1990s, Ning et al. conducted in vitro cell experiments to determine the ability of LND to increase the killing of several cytotoxic drugs against murine RIF-1 and human HT1080 fibrosarcoma cells [50]. It was demonstrated that LND enhanced the cytotoxicity of these agents; however, the enhancement of cytotoxicity was related to the sequence of drug combination. Unlike other drugs (e.g., CDDP, bleomycin, and mitomycin C), the maximum potentiation of BCNU toxicity was seen when LND was administrated (24 h) prior to BCNU exposure (1 h). As an acidic environment can inhibit MGMT, resulting in reduced DNA repair, we speculate that the acidic environment induced by LND inhibits MGMT and potentiates the cytotoxicity of BCNU. Whether other nitrosoureas in combination with LND also show the greatest cytotoxicity when LND precedes nitrosoureas is still to be explored. However, in a following in vivo study, the results showed that the combination of LND and BCNU was not significantly more active than the single BCNU against mice RIF-1 tumor [35]. Further research is needed to investigate the combined effect of LND and BCNU or other nitrosoureas and the effect of dosing sequence on cytotoxicity.

### 2.5. LND in Combination with Anthracyclines

Anthracyclines (ANTs), such as doxorubicin (DOX) and epirubicin (EPB), are commonly used for treating malignant tumors in clinic [79,80]. Their anticancer activity is associated with the ability to form special DNA adducts, thereby inhibiting DNA replication and RNA transcription [74]. In 1992, a paper reported that LND increased the cytotoxic effect of DOX in primary and established breast cancer cell lines [52]. However, the probable chemosensitizing mechanisms were not investigated in this study. In a following study, LND was proven to enhance the DOX content in resistant ascites tumor cells via modulating energy metabolism [53].

As mentioned above, most tumors exhibited a slightly more acidic pHe than pHi. Nath et al. also proposed that the tumor pH gradient of the plasma membrane had an important role in maintaining the intracellular concentration and activity of chemotherapeutic drugs, as most anticancer drugs enter tumor cells by free diffusion, which would contribute to the uptake of weak acids in comparison with weak bases [54]. In this case, weak bases outside the cells would be transformed into the charged protonated ammonium form, which cannot be diffused into the cell [54]. LND is known to selectively cause intracellular acidification and deplete cellular energy within tumors [76]. Furthermore, LND-induced decrease of pHi was much more obvious than pHe [54], this would lead to the high uptake of weak basic DOX by tumors, which is due to the cation trapping mechanism as a result of protonation driven by the enhanced intracellular acidification [55]. In LND-treated mice xenografts of human DB-1 melanoma, pHi decreased from 6.90 to 6.33 meanwhile pHe dropped only from 7.0 to 6.80 [42]. The bioenergetic state of the tumor, expressed as β-NTP/Pi ratio, also decreased by 66.8% [42]. Similarly, in several mice xenograft models of human breast, prostate, and ovarian cancer, LND also produced intracellular tumor acidification and ATP depletion [54]. Based on the previous study that LND potentiated the response of DB-1 melanoma model to melphalan [10], Nath et al. demonstrated LND increased the sensitivity of both DB-1 melanoma and HCC1806 breast carcinoma to the killing by DOX, in which LND in combination with DOX yielded significant tumor growth delays compared to DOX or LND alone [54]. LND may enhance DOX activity in two ways: (1) LND induced intracellular acidification and reduced the pHi. Thus, the pH gradient of tumor was reversed so that free basic DOX (pKa ~8.5) would be more readily captured in the acidified tumor cells (cation trapping) [54]; (2) for another, similar to TMZ and NM, LND-mediated tumor de-energization inhibits energy-dependent MDR efflux pumps, resulting in more DOX retained in tumor cells. In 2018, Nath et al. reported the combined effect of LND plus DOX against two different human melanoma cell lines (DB-1 and WM983B) [56]. It was found that WM983B melanoma was less glycolytic than DB-1, however, intracellular acidification and de-energization were also induced after exposure to LND in immunosuppressed mice WM983B xenografts. It was not surprising that the tumor growth delay for DB-1 melanoma (25.4 days) was more obvious than that of WM983B (13.7 days) after the combination treatment of LND plus DOX, because WM983B melanoma cells are less energetic than DB-1 cells, LND-mediated energy depletion should be less in WM983B tumors, thus being less effective in increasing the activity of DOX. Recently, Jin et al. constructed an all-in-one multifunctional pH-sensitive tumor-targeted delivery system, where LND is used as a chemosensitizer and DOX as a chemotherapeutic agent were integrated into a micelle [81]. The unique pH-sensitive DOX/LND micelle displayed effective targeting to tumor sites, resulted in burst release of DOX under acid tumor microenvironment (~pH 6.5). In vivo studies indicated that the micelle had enhanced antitumor effects and decreased off-target effects against Skvo3 ovarian cancer xenograft models. More importantly, the novel micelle system reduced the DOX-induced cardiac fibrosis and hematological toxicity. Further studies showed that the underlying mechanisms of better therapeutic effects include (1) LND-induced ATP deprivation decreases the supply of energy for MDR efflux pumps, more drugs (DOX) were retained in tumor cells, (2) mitochondria-dependent apoptosis, (3) antiangiogenic effect, and (4) effective killing of cancer stem cells [81].

In addition to DOX, LND can also be combined with other ANTs to exhibit better anticancer activity. For example, Nistico et al. reported a clinical trial that 51 patients with advanced breast cancer were treated with a combination of LND and EPB. The results suggested the combination of LND and EPB was effective for the treatment of advanced breast cancer [57].

Similar to the Pt(IV)-ADD prodrugs described above [18], Li and co-workers coupled ADD and DOX into a single chemical entity (DOX-ADD conjugate) through an acid-sensitive hydrazone bond, then encapsulated in a nanocarrier DSPE-PEG_2000_ micelles for co-deliver of DOX and ADD [19]. The novel drug formulation DOX-ADD(M) was stable under normal physiological conditions, but rapidly broken to release individual drugs within acidic environment. Through endocytosis, DOX-ADD(M) was internalized into drug-resistant MCF-7/ADR cancer cells and existed in mildly acidic endolysosomes. DOX-ADD(M) showed more potent anti-MDR ability when compared to free ADD–DOX conjugates, which could be due to the increased hydrolysis rate of ADD-DOX conjugates in endolysosomes, followed by the release of DOX and ADD. After that, based on the principle of “molecular economy”, a DOX-ADD@TPGS-NO micelle system was constructed, in which each component (DOX, ADD, TPGS, and NO) could have at least one function [82]. DOX acts as the main cytotoxic agent, ADD and TPGS serve as mitochondria-inhibiting agents to reverse MDR by ATP depletion, and NO is used to improve drug penetration and accumulation in tumor by normalizing tumor vasculature and enhancing oxygenation level. The DOX-ADD@TPGS-NO micelles had controlled drug release with obvious pH-sensitive property, high cellular uptake, significant cytotoxicity, increased tumor accumulation, significant tumor growth inhibition and great potency in inhibiting lung metastasis of murine mammary carcinoma. Furthermore, the DOX-ADD@TPGS-NO micelles exhibited good safety as indicated by no obvious pathological sites found in main organs of mice [82]. Recently, Wang et al. also reported pH-sensitive Aa-DOX+ADD@PC nanoparticles with more precise DOX and ADD ratiometric control, where PC was poly(β-aminoester)-*g*-(β-cyclodextrin) (PBAE-*g*-β-CD) copolymers, 1-adamantaneacetic acid (Aa) was conjugated with DOX (Aa-DOX) and then encapsulated via host–guest interactions between the adamantyl group and β-CD, and Aa-TPGS as a hydrophilic moiety was used to increase the stability of the nanoparticles [83]. Results suggested the nanoparticles showed an effective inhibition towards drug-resistant MCF-7/ADR cells in vitro and in vivo [83].

### 2.6. LND in Combination with 6-Diazo-5-oxo-l-norleucine

In addition to enhanced glycolysis, the increased pentose phosphate pathway (PPP) flux, the high rates of glutaminolysis, and the synthesis of fatty acids (FAs) are also the malignant metabolic phenotypes of cancer cells [58,84,85]. When glycolysis is inhibited, glucose metabolism is impaired; however, tumor cells can survive by utilizing the glutaminolysis as a complementary surviving pathway, and vice versa [56,58]. Therefore, it is considered to kill cancer cells through the combined inhibition of malignant metabolic pathways.

**Figure 3:**
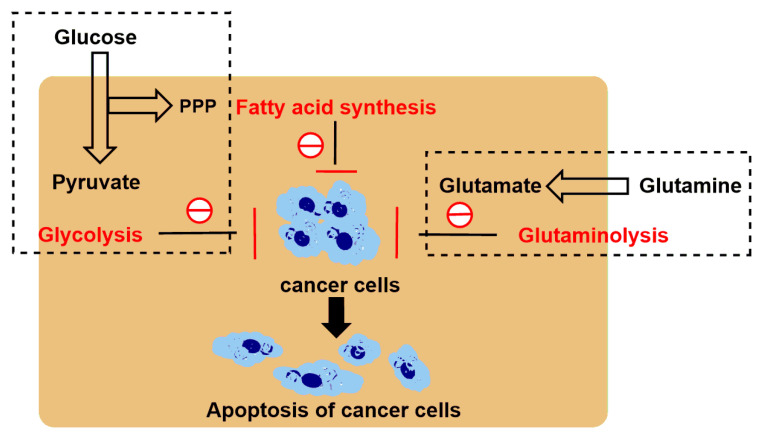
Targeting three key ways to cut off the tumor’s energy and matter supply. Cancer cells obtain energy and matter mainly through glycolysis, glutaminolysis, and fatty acid synthesis. Cutting off these three pathways can inhibit tumor growth.

**Figure 4:**
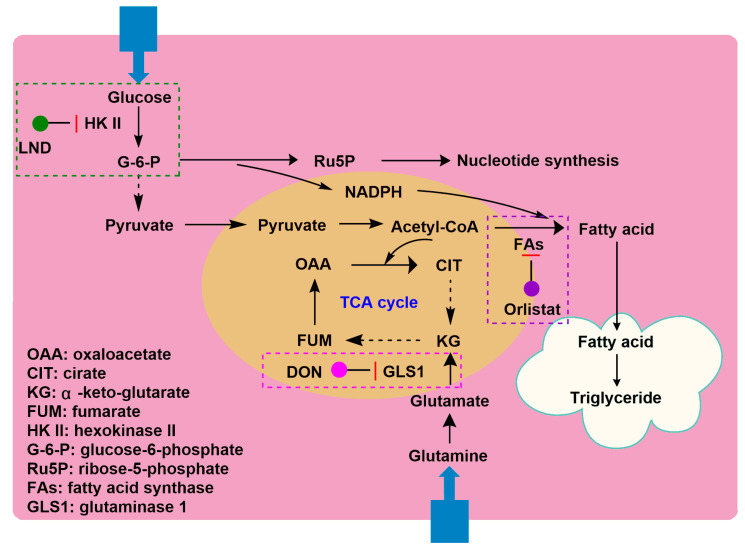
Synergistic model of DON in combination with LND and orlistat. LND inhibits glycolysis by inhibiting hexokinase II (HK-II), DON inhibits glutaminolysis by inhibiting kidney-type glutaminase (GLS1), and Orilistat inhibits fatty acid synthesis by inhibiting the FA synthase.

As mentioned above [56], we have discussed the sensitivity of two different melanoma models to the combination of LND plus DOX. Compared to DB-1 melanoma xenograft, WM983B melanoma xenograft responded less to DOX [56]. Of note, after LND treatment, WM983B melanoma xenografts upregulated glutaminolysis to compensate the energy deprivation caused by LND-mediated MPC inhibition [56]. The high rate of glutaminolysis provides a readily available source of carbon backbone and nitrogen that promotes tumor growth [86]. The tumor can metabolize glucose and glutamine in coordination and survive under conditions of malnutrition and hypoxia [3]. For example, oncogene *K-ras* can enhance glycolysis activity, decrease oxidative flux through the tricarboxylic acid cycle, and leading to an increase in the utilization of glutamine for anabolic synthesis [87]. When cancer cells were treated with LND, a significant increase in glutamine flux was observed by isotopic labeling, as well as a significant increase in the production of ammonia metabolites succinic acid, fumaric acid, malate, and citric acid. This indicates that when the glycolysis is inhibited, the metabolic pathway begins to shift towards glutamine [3]. Therefore, the dual inhibition of both glycolysis and glutaminolysis may be one of the promising strategies for increased anticancer efficacy. 6-Diazo-5-oxo-l-norleucine (DON), as an analog of glutamine, is often used as an inhibitor of glutaminolysis [59]. In addition, in view of the experience with DON in cancer treatment, it can be found that DON is a relatively safe candidate drug, whose dose-limiting toxicity is nausea and vomiting that vary upon the schedule and dose. Nowadays, this problem should be dealt well because of the availability of potent and effective antiemetics [58]. As a result, it is rational to use the combination of LND and DON for simultaneously blocking glycolysis and glutaminolysis as anticancer therapy. In early studies, DON was used in combination with antimetabolites to treat murine mammary tumors [88]. Over the past several decades, there have been few studies in which glycolytic inhibitors and DON have been combined to treat tumors; except for a study reported in 1993, in which the combination of 2-deoxyglucose (2-DG, a glycolytic inhibitor) and DON increased growth inhibition in human myeloid leukemia cells [89], no related studies we found until the works reported in recent years by Cervantes-Madrid’s group [7,8,58]. They demonstrated that the combination of LND and DON plus orlistat [90] (a FA synthase inhibitor) targeting three key pathways (glycolysis, glutaminolysis, and de novo synthesis of FAs), increased killing effects in 13 cancer cell lines in comparison with primary fibroblasts [7] (Figure 3 and Figure 4). The synergistic effect of the triple combination was observed in colon cancer cell line SW480. Moreover, the clinical feasibility of the triple combination was reflected by the well tolerance when administered to healthy BALB/c mice. In a following in vivo study, Cervantes-Madrid and co-workers demonstrated the antitumor efficacy of triple combination in a colon cancer CT26.WT syngeneic tumor model in BALB/c mice and a human colon cancer SW480 xenograft tumor model in nude mice [8]. Especially, no evident toxicity occurred and mice weight was maintained during treatment.

### 2.7. LND in Combination with Arsenic Trioxide

**Figure 5:**
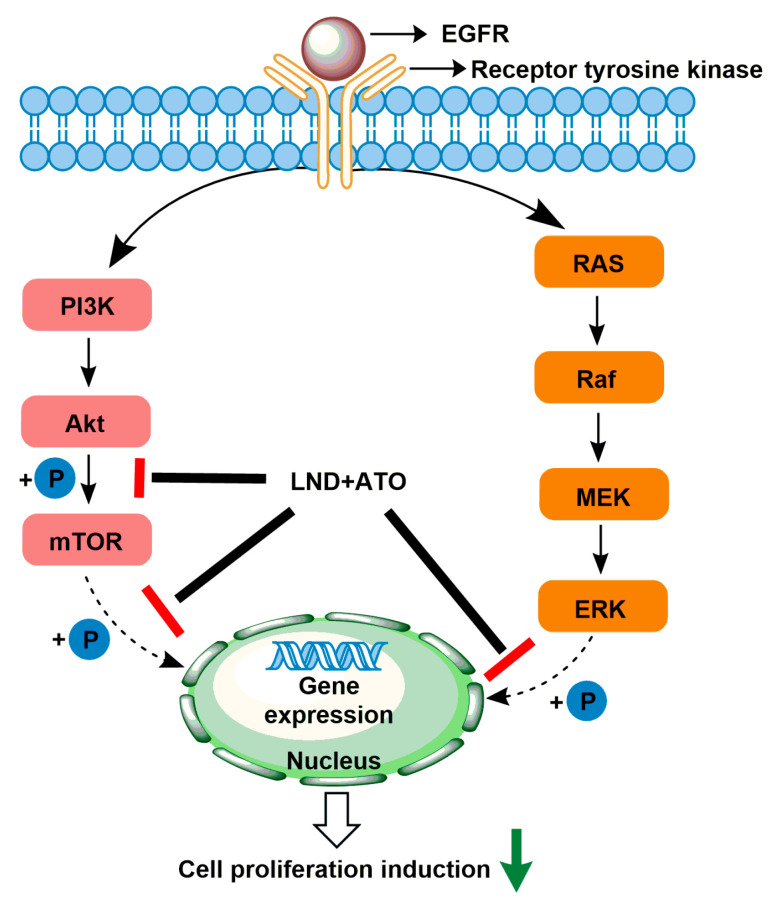
Synergistic model of the combination of ATO and LND. The combination of ATO and LND could inhibit the phosphorylation of AKT, mTOR, and ERK in the MEK/ERK and AKT/mTOR signaling pathways, respectively, and reduce cell proliferation.

As a clinical anticancer drug, arsenic trioxide (ATO) is effective in the treatment of acute promyelocytic leukemia (APL). Because many biochemical actions (apoptosis, growth inhibition, angiogenesis, and differentiation) are involved in mediating the anticancer effect of ATO, it is potentially synergistic with other agents to achieve increased therapeutic efficacy not only in APL, but also in other hematologic cancer and solid tumors [61]. ATO-mediated ROS production plays a crucial role in mediating the anticancer effect; the GSH-based redox system is the one determinant of tumor chemosensitivity to ATO [61]. Generally, ATO activity is inversely proportional to the content of intracellular GSH in tumor cells [62,63]. In this case, the prooxidant action of LND through the inhibition of mitochondrial respiratory chain that can lead to the generation of ROS may enhance the killing efficacy of ATO as an anti-leukemic agent. Calvino et al. demonstrated that LND plus ATO induced increased apoptotic efficacy in human leukemia cells via ROS generation and the modulation of defensive protein kinase signal pathways (MEK/ERK and AKT/mTOR) (Figure 5) [64]. The authors found that mitochondrial dysfunction was involved in the apoptosis induced by the combination of LND and ATO, as indicated by early mitochondrial PT pore opening and late mitochondrial transmembrane potential dissipation. The activation of the intrinsic apoptotic pathway was indicated by multiple apoptotic events, including Bcl-X_L_ and Mcl-1 downregulation, Bax translocation to mitochondria, mitochondrial release of cytochrome c and Omi/HtrA2 release to the cytosol, XIAP downregulation, and caspase-3/-9 activation, and the secondary activation of caspase-8/Bid axis [64]. Interestingly, in this study, the authors found LND caused the activation of ERK and AKT/mTOR pathways that was evidenced by increased phosphorylation of ERK, AKT, p70S6K, and rpS6. The activation effects were decreased by the addition of the MEK/ERK, PI3K/AKT or mTOR inhibitors. This may partly explain the low efficacy when LND was used in monotherapy. However, ATO inhibited the activation of these signaling pathways, justifying the synergistic anticancer effects of ATO and LND (Figure 5) [64]. The underlying synergistic mechanisms between LND and ATO include (1) ATO attenuates LND-mediated activation of MEK/ERK and AKT/mTOR defensive pathways and (2) LND-induced ROS production enhances the apoptosis-inducing capacity of ATO in cancer cells.

### 2.8. LND in Combination with Curcumin

Curcumin (CCM), extracted from the rhizome of turmeric, is a hydrophobic plant polyphenol. It has antioxidant and anti-inflammatory properties and has been demonstrated to be an effective chemoprotective agent [66,91]. Besides, CCM also has anticancer activity. Previous studies showed that CCM-induced cancer cell apoptosis involved multiple targets and signaling pathways, including increasing the permeability of mitochondrial membrane (PT pore opening), downregulation of antiapoptotic proteins Bcl-2, Bcl-X_L_, and XIAP, upregulation of proapoptotic proteins Bax and Bak, caspase-3 activation, the release of cytochrome c, as well as the inhibition of PI3K/AKT and NF-κB survival pathways [66,67]. Furthermore, it is of great interest to note that CCM-induced apoptosis is associated with the ROS generation in tumor cells, in spite of it acts as an antioxidant [66]. In consideration of CCM and LND are both mitochondrial-targeting agents, and have similar mechanism of action, such as mitochondria dysfunction and ROS production, their combination may have cooperative anticancer effects. Indeed, Sanchez et al. showed that when used in combination, sub-cytotoxic CCM doses (7.5 μM) greatly increased the apoptosis induction by LND in human U937 acute myeloid leukemia (AML) cells, compared to each drug alone [67]. They pointed out that the ROS induction played a pivotal role, as indicated by the co-treatment with the antioxidants (e.g., *N*-acetyl-l-cysteine (NAC)) significantly reduced apoptosis, mitochondrial membrane potential dissipation and cytochrome c release by the combination of CCM and LND, while the co-treatment with the pro-oxidant agents (e.g., H_2_O_2_ and 2-methoxyestradiol) potentiated the apoptosis. After exposure to CCM plus LND, a series of biological events closely associated to apoptosis were observed, such as proapoptotic Bax and Bid activation, cytochrome c release from mitochondria to cytosol, downregulation of XIAP expression, and caspase-3/-9 activation [67]. Thus, co-treatment with CCM may be a useful strategy to enhance the efficacy of LND in human myeloid leukemia as well as other solid tumors, however, more and more studies are needed to verify the feasibility.

### 2.9. LND in Combination with Matrine

**Figure 6:**
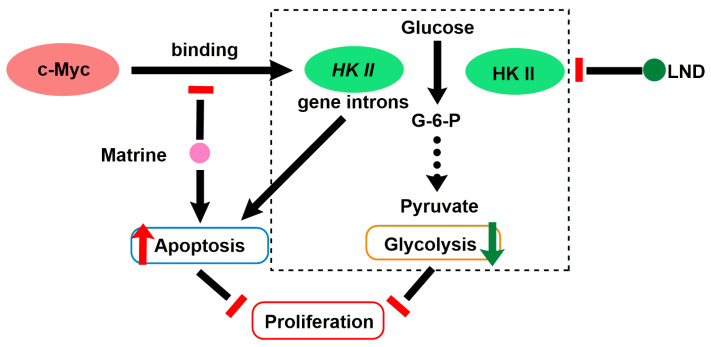
Synergistic model of the combination of MAT and LND for the treatment of human myeloid leukemia cells. MAT can inhibit the binding of c-Myc to *HK-II* gene intron. When in combination with LND, it can reduce the expression of HK-II and inhibit the glycolysis.

Matrine (MAT) is an alkaloid compound isolated from Chinese traditional medicine radix *Sophora flavescens* and has anticancer effect on multiple carcinoma diseases, such as hepatoma, lung cancer [92], gastric cancer [93], and human myeloid leukemia [94]. In recent years, it has been reported that MAT can regulate the cycle distribution of tumor cells so as to inhibit the proliferation of tumor cells [68], or increase the efficacy of chemotherapeutics, thus reducing the dosage and adverse effects of chemotherapeutic agents [95]. Lin et al. demonstrated that MAT induced apoptosis in human myeloid leukemia cells through the downregulation of *HK-II* mRNA expression mediated by reduction in c-Myc binding to *HK-II* gene intron, leading to the downregulation of HK-II protein. They further found that expression of proapoptotic protein Bad was inversely correlated with the expression level of HK-II in K562 stable cell lines treated with MAT (Figure 6) [69]. HK-II knockdown/overexpression promoted/inhibited MAT-induced apoptosis and growth inhibition in K562 myeloid leukemia cells, respectively. Thus, in order to examine if pharmacological inhibition of HK-II could sensitize human myeloid leukemia cells to MAT, they selected LND as HK-II inhibitor to combine with MAT. As expected, in both K562 and HL-60 cells, co-treatment with MAT and LND showed significant growth inhibitory effect in comparison with MAT or LND alone. The synergetic effect of MAT and LND was observed in both K562 and HL-60 cells in vitro, as well as in tumor-bearing mice of K562 cells in vivo [69]. The synergetic mechanism is mainly because that they both downregulate or inhibit HK-II expression, which inhibits the glycolysis and causes apoptosis of tumor cells (Figure 6). This study proposed a new idea to improve the therapeutic efficacy of MAT by combining with LND in future clinical application.

## 3. LND Sensitizes Tumor Cells to Physical Therapy

After exposure to chemotherapeutics, cell survival can be modified by the post-exposure environment. Therefore, oncologists and pharmacologists are trying to treat cancer using new therapies combining chemotherapeutics other than traditional chemotherapeutics alone. LND as a traditional anticancer candidate drug; it is capable of sensitizing tumors to physical therapy. The following section is a brief overview of LND in combination with radiotherapy (RT), hyperthermia (HT), and photodynamic therapy (PDT) (Table 2).

### 3.1. LND Sensitizes Tumor Cells to Radiotherapy

Radiotherapy (RT) is able to cause the disruption of DNA replication, thus leading to cell death. Normal cell population has poor sensitivity to radiation, strong tolerance and high repair ability, while the tumor tissue is opposite [96]. It has been shown that high level of repair ability for radiation-induced potentially lethal damage (PLD) may cause the radioresistance of human tumors and PLD repair is an energy-dependent process [110]. Surprisingly, it is clear that few chemotherapeutics can inhibit the recovery after experiencing X-irradiation treatment [111]. In this case, LND, an energy blocker, may be capable of inhibiting the PLD repair, thus enhancing the efficacy of RT. Previous studies showed that the combination of LND and RT delayed tumor cell growth in vitro and in vivo, meanwhile without obvious effects to normal tissues [97,98,99]. Hahn et al. found that ~150 μM LND completely abolished the recovery from PLD in Chinese hamster cells (HA-1) after exposure to X-irradiation [97]. Even a lower concentration of LND (~30 μM) was sufficient to completely inhibit the cell survival when LND was present before, during and after irradiation [97]. The doses of LND are readily and safely achievable in humans. Later, Kim et al. proved that LND enhanced the effects of radiation on two mouse tumor models (methylcholanthrene-induced fibrosarcoma in BALB/c mice and radiation-induced fibrosarcoma in C3H/He mice), as indicated by increased tumor growth delays and local tumor control rates [98]. The maximal radiosensitizing effect was obtained when LND was administered immediately prior to or after irradiation. The theoretical basis for the potentiating effects may be that LND interferes with the repair of radiation-induced PLD, as mentioned above. Under a lower dose of radiation, LND’s radiosensitizing effect seems to be more effective, which is very important in clinical setting because most radiation regimens consist of fractionated treatments given over several weeks. Subsequently, in multicellular tumor spheroids (MTS) of HeLa cells, Kim and co-workers also demonstrated that LND potentiated the radiation effects, as shown by the enhancement in MTS growth inhibition [99]. It is worth noting that no an apparently comparable increased skin reaction was observed following the combined treatment of radiation and LND, indicating the possible low toxicity to normal tissues [98].

### 3.2. LND Sensitizes Tumor Cells to Hyperthermia

Hyperthermia (HT) refers to physical heating that is to achieve the goal of killing tumor cells. Owing to abnormal vascular growth and structural disorder in tumor tissues, capillaries are compressed with the formation of blood sinuses, and, after being heated, the tumor becomes a heat reservoir, with a 3–5 °C higher temperature than that of adjacent normal tissues [100]. This selective heating leads to temperature difference between tumor and normal tissues, which ensures that when the tumor is heated to 40–43 °C, most of tumor cells can be killed, without damaging normal tissue cells, resulting in tumor selective HT [101]. Currently, the precise mechanisms of HT remain unknown; however, several possible opinions were proposed [100]: (1) destruction of tumor cell membrane, leading to the enhancement of permeability; (2) deformation of cytoskeleton; (3) inhibition of DNA synthesis; and (4) impaired tumor vasculature. In addition, HT can induce heat shock proteins (HSPs), results in the improvement of immunity. HSPs are highly expressed in cells, up to 1% of the total intracellular proteins. In tumor cells, the expression is much higher, up to about 6–7%. Therefore, inhibition of HSPs expression can also make tumors sensitive to heat [112]. The unique characteristics of HT and its pleiotropic effects are favorable for its combination with chemotherapy.

As described above, LND has a weak cytotoxicity against various tumor cells; however, its anticancer activity can be enhanced by HT [101]. Ultrastructural observations performed by Silvestrini et al. showed that heating led to the formation of condensed mitochondria in tumor cells in vivo, meanwhile condensed mitochondria is probably a specific target of LND [102]. They found that LND sensitized tumor cells to HT in vitro and in vivo. It was expected that under the treatment of LND plus heating, severe mitochondrial damage was observed [102]. In a following study, Silvestrini’s group demonstrated that LND was a potent HT sensitizer of HeLa cells in vitro and methylcholanthrene-induced fibrosarcoma in BALB/c mice [101]. It should be noted that the potentiation of thermal sensitization by LND was dependent on extracellular pH and increased under the low pH condition [101]. The pH-dependent thermal sensitization by LND was also proven by Raaphorst et al. in human glioma and head-neck squamous cell carcinoma cells [15], as well as by Coss et al. in human melanoma DB-1 cells [103]. In the latter study, LND exposure reduced pHi of melanoma cells under tumor-like pHe, so as to abrogate the induction of HSPs at HT, leading to thermal sensitization [103,104]. On the contrary, at a normal tissue-like pHe (7.3), LND didn’t sensitize cells to heat treatment [103]. Another rational explanation for pH-dependent thermal sensitization may be that, under more acidic extracellular pH, LND, a weak acid, is more concentrated in the relatively more basic intracellular compartment. Particularly, the acidic microenvironment of solid tumor is in favor of the thermal sensitization mediated by LND in the clinical setting. Another study performed in the Dunning R3327G rat prostatic adenocarcinoma indicated that LND could significantly reduce the heat dose without the compromise of the anticancer efficacy, and avoid the HT-induced complications [105].

Overall, the mechanisms of the thermal sensitization mediated by LND may include [100] (1) heating can lead to the formation of condensed mitochondria, which LND targets more easily; (2) HT increases blow flow and drug delivery; (3) increased drug uptake, HT increases cell permeability, allowing more chemotherapeutic drugs to enter the cells; and (4) LND decreases the intracellular ATP level and induces intracellular acidification, thus inhibiting the repair of HT-induced lethal or sublethal damages as well as the proteins involved in cell survival.

### 3.3. LND Sensitizes Tumor Cells to Photodynamic Therapy

Photodynamic therapy (PDT) is based on the photochemical reactions among three main components: photosensitizers, light with appropriate wavelength, and oxygen inside the cells [106]. Generally, after entering corresponding pathological tissues, a photosensitizer is excited under the irradiation with a specific wavelength; then, the photosensitizer in excited state transmits the energy to the oxygen molecules to generate highly reactive singlet oxygen, followed by the oxidative damage of biomacromolecules and producing cytotoxic effects, leading to cell damage and even death [106]. So far, PDT has been used to diagnose and treat tumors in many hospitals. Recent studies have illustrated that the combination of PDT and other treatments, including chemotherapy, can greatly improve the therapeutic effect, because the combination-based mechanisms can effectively overcome tumor resistance [113]. The response of tumor cells to PDT mainly depends on the subcellular localization of photosensitizer and the production of ROS [114,115]. In fact, PDT causes mitochondrial damage and inactivates several glycolytic enzymes leading to a significant decrease of intracellular ATP level, thereby inducing cytotoxicity [107]. Therefore, it may be feasible that the inhibitors of energy metabolism are able to enhance the sensitivity of tumors to PDT. Based on this assumption, LND as a blocker of energy metabolism was combined with 5-aminolevulinic acid (ALA), a classical photosensitizer in clinical applications, to potentiate the effects of ALA-mediated PDT [108]. When LND was administrated prior to light exposure, a synergistic effect of ALA-mediated PDT and LND at non-toxic doses was achieved [108]. In 2013, Golding et al. reported that the addition of LND significantly increased the cytotoxicity of ALA-mediated PDT by 10-fold in human breast cancer MCF-7 cells using a clone formation assay [109]. In contrast, glycolysis inhibition by LND only slightly enhanced the PDT cytotoxicity in normal primary human mammary epithelial cells (<10% cell death), suggesting LND selectively increased PDT cytotoxicity in cancer cells. The intracellular PDT-induced ROS level was also increased after LND exposure. However, the authors reminded that only LND was added after ALA treatment, the potentiation of PDT cytotoxicity occurred, because if co-treatment LND inhibited the intracellular accumulation of photosensitizer ALA. It is of great interest to note that in a recent study, a multifunctional nanomedicine system (AS-LAGN) was constructed by gold nanoparticles (GNP) conjugated with LND, albumin (BSA), and aptamer AS1411, in which GNP exerts photothermal property, LND acts as mitochondria-targeting drug, BSA improves bioavailability and aptamer AS1411 has tumor-targeting property [116]. Results indicated that the AS-LAGN nanomedicine exhibited a synergistic photothermal chemotherapy for tumor ablation in animal models, meanwhile without obvious damage to surrounding normal healthy tissues. In fact, this study also belongs to the combination of LND plus HT. Although the limited reports or studies, the combination of PDT and LND is worthy of further study in the clinical setting, especially for the cancers having been recommended using PDT.

## 4. Conclusions

Existing chemotherapeutics often face drug resistance in a clinical setting. Accordingly, applying the combination of existing chemotherapeutic drugs or therapeutic methods with different mechanisms of action, to some extent, can effectively overcome tumor resistance. LND has been employed in recent clinical trials in combination with other chemotherapeutic agents based on the characteristics of its low toxicity and targeted energy metabolism. By discussing the synergistic mechanisms of LND with the aforementioned nine chemotherapeutic agents and three physical therapies, it is suggested that LND increases the anticancer effects of chemotherapeutic agents or physical therapies primarily for five reasons. First, LND selectively reduces the intracellular energy of tumor cells via inhibiting glycolysis and mitochondrial respiration, thereby interfering with energy-dependent DNA damage repair and preventing the drug efflux by energy-dependent MDR pump, respectively. Second, LND induces intracellular acidification via the inhibition of lactic acid efflux to inactivate the activity or expression of related proteins. One hand, a more acidic intracellular environment inhibits the expression of MGMT enzyme thus affecting the repair of damaged DNA, and abrogates the induction of HSPs at HT. On the other hand, intracellular acidification is capable of reversing the pH gradient, thereby expediting weakly basic chemotherapeutic drugs to enter the cancer cells. Third, LND-induced ROS production can increase the activity of drugs sensitive to oxidative stress. Fourth, LND is capable of impacting apoptosis-related proteins (e.g., suppressing the expression of antiapoptotic Bcl-2, promoting the proapoptotic Bax expression, and exerting a synergistic effect with drugs that induce the expression of apoptotic proteins). Fifth, LND decreases the cellular levels of GSH and NAPDH, down-regulates the PPP, leading to the inhibition of GSH-mediated drug inactivation and the materials supply for cancer cell proliferation. Currently, the research on LND in the past five years has not only focused on its combination with other drugs, but also improved its targeting ability to tumors through coupling with tumor targeting agents, as well as nanomedicine. In summary, it should be noted that multiple biological effects, tumor selectivity, and low toxicity to normal tissues are the critical characteristics that make LND a promising potentiator to improve the therapeutic effects of anticancer drugs or physical therapies in clinical cancer treatment. We are looking forward to the emergence of more elaborative and systemic studies in terms of the combination of LND with chemotherapy or physical therapy. This is the future direction of our laboratory.

## Figures and Tables

**Table 1:** Combination of LND with nine chemotherapeutic agents.

Name	Structure	Mechanisms of Action	Tumor Type	Synergistic Mechanism	Adverse Effect	Ref.
Cisplatin (CDDP)	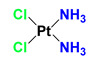	CDDP induces DNA cross-linking to destroy the DNA structure.	MCF-7, MX-1 breast carcinoma cells (in vivo); A2780, IGROV-1 ovarian carcinoma cells (in vivo); NSCLC cells (in vivo); LNCaP cells (in vivo)	(1) Synergistically induces downregulation of Bcl-2, decrease antiapoptotic activity of cancer cells. (2) LND interferes with DNA repair process for the DNA damages induced by CDDP.	Myelosuppression; Nephrotoxicity; Neurotoxicity; Gastrointestinal reaction	[31,32,33,34,35,36]
Temozolomide (TMZ)	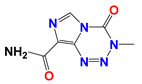	TMZ degrades to generate active MTIC, which is converted to methyl diazonium ion, followed by alkylation on the O^6^ or N^7^ site of guanine.	DB-1 melanoma xenografts (in vivo)	LND inhibits the MDR efflux of TMZ through the reduction of the bioenergy state of the tumor.	Myelosuppression; Neurotoxicity; Thrombocytopenia; Granulocytosis	[37,38,39]
Nitrogen mustards (NMs)	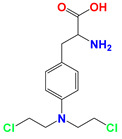 Melphalan	NMs can form electron-deficient ethyleneimine ions, and then covalently bind nucleophile groups (electron-rich) of biomacromolecules for alkylation, resulting in DNA intrastrand and interstrand cross-linking.	DB-1 melanoma xenografts (in vivo)	(1) LND induces intracellular acidification, leading to increased concentration of the active aziridinium ion intermediate that yields DNA damage. (2) LND induces de-energization prevents the energy-dependent MDR pump from pumping out the drugs. (3) LND-induced intracellular acidification inhibits GST activity, leading to decreased level of GSH which can quench the active aziridinium species. (4) DNA repair is reduced by acid inhibition of repair proteins.	Myelosuppression; Nephrotoxicity; Gastrointestinal reaction	[40,41,42,43,44,45,46]
	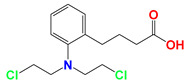 Chlorambucil					
	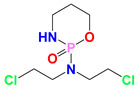 Cyclophosphamide					
	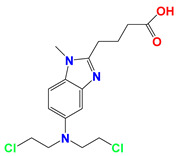 Bendamustine					
Carmustine (BCNU)	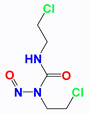	BCNU induces DNA guanine O^6^-alkylation, followed by the formation of DNA interstrand cross-linking	HT1080 human fibrosarcoma cells (in vitro); Murine RIF-1 fibrosarcoma cells (in vivo)	LND induces acidic environment that may inhibit MGMT and potentiates the cytotoxicity of BCNU.	Myelosuppression; Vascular toxicity; Neurotoxicity; Liver impairment; Lung toxicity	[35,47,48,49,50]
Anthracyclines (ANTs)	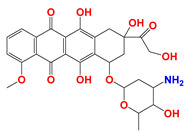 Doxorubicin	ANTs form special DNA adducts, thereby inhibiting DNA replication and RNA transcription.	Breast carcinoma cells (in vivo); WM983B, DB-1 melanoma xenografts (in vivo); Ehrlich ascites tumor cells (in vitro); H2030 cells (in vivo); H2030BrM3 cells (in vivo)	(1) LND inhibits the MDR efflux of ANTs through reducing the bioenergy state of tumors. (2) LND reverses pH acidity gradient, leading to high uptake of free basic DOX by tumors.	Myelosuppression; Cardiotoxicity	[51,52,53,54,55,56,57]
	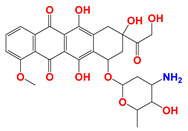 Epirubicin					
6-Diazo-5-oxo-L-norleucine (DON)	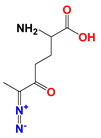	DON inhibits glutaminolysis	HCC1806 breast carcinoma cells (in vitro); HeLa cells (in vitro); A375 melanin cells (in vitro); SW480 colon cancer cells (in vitro)	Combined with LND and orlistat, blocking three key pathways: glycolysis, glutaminolysis and fatty acid synthesis, achieve synergistic killing of cancer cells.	Myelotoxicity; Oral mucositis; Uremia; Gastrointestinal reaction	[7,8,58,59]
Arsenic trioxide (ATO)	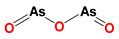	(1) ATO induces PT pore opening and activates intrinsic apoptotic pathways. (2) ATO induce ROS production.	HL60 acute myelocytic leukemia cells (in vitro)	(1) ATO attenuates LND-mediated activation of MEK/ERK and AKT/mTOR defensive pathways; (2) LND-induced ROS production enhances the apoptosis-inducing capacity of ATO in cancer cells.	Hepatotoxicity; Cardiotoxicity	[60,61,62,63,64]
Curcumin (CCM)	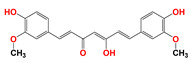	CCM induces PT pore opening and the inhibition of defensive signaling pathway such as PI3K/AKT and NF-κB.	U937 acute myelocytic leukemia cells (in vitro)	CCM and LND synergistically destroy mitochondrial membrane structure and induce ROS production.	Gastrointestinal reaction	[65,66,67]
Matrine (MAT)	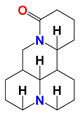	(1) MAT reduces the expression of cell cycle protein mRNA in G_0_/G_1_ phase to block the cell cycle. (2) MAT reduces c-Myc binding to *HK-II* gene introns.	HL60 acute myelocytic leukemia cells (in vitro); K56 chronic myelocytic leukemia cells (in vivo)	MAT and LND synergistically downregulate or inhibit HK-II expression.	Degeneration of nerve cells in brain tissue	[68,69]

**Table 2:** Combination of LND with physical therapy.

Therapy Method	Mechanism of Action	Tumor Type	Synergistic Mechanism	Ref.
Radiotherapy (RT)	Cancer cells exhibit sensitivity to radiation, poor tolerance, while the normal cell population is opposite	BALB/c, C_3_H/He mice fibrosarcoma cells (in vivo); HeLa cells (in vivo)	LND interferes with the energy-dependent PLD repair process.	[96,97,98,99]
Hyperthermia (HT)	Cancer cells can easily store more heat than normal cells. When heated to 40–43 °C, cancer cells undergo membrane structure destruction, cytoskeleton deformation, DNA synthesis inhibition, and blood vessel damage, thus resulting in death.	BALB/c mice fibrosarcoma cells (in vivo); HeLa cells (in vivo); human glioma cells (in vivo); Head-neck squamous cells (in vivo); DB-1 melanoma xenografts (in vivo); R3327G rat prostatic adenocarcinoma (in vivo)	(1) HT can lead t78o the formation of condensed mitochondria, which LND targets more easily. (2) HT increases blow flow and drug delivery. (3) HT increases cell uptake of drugs. (4) LND inhibits the repair of HT-induced (sub)lethal damage as well as the proteins involved in cell survival.	[100,101,102,103,104,105]
Photodynamic therapy (PDT)	PDT can cause mitochondrial damage, causing ATP consumption and ROS production.	MCF-7 human breast carcinoma cells (in vitro)	LND and PDT synergistically destruct mitochondrial structure, decrease intracellular ATP level, and induce the generation of ROS.	[106,107,108,109]
